# Supplementary material for: Communication between DNA polymerases and Replication Protein A within the archaeal replisome
Source: Nat Commun. 2024 Dec 30;15:10926. doi: 10.1038/s41467-024-55365-w (PMC11686378; doi:10.1038/s41467-024-55365-w)
Supplement: Supplementary file 1 — Supplementary Information [file 41467_2024_55365_MOESM1_ESM.pdf]

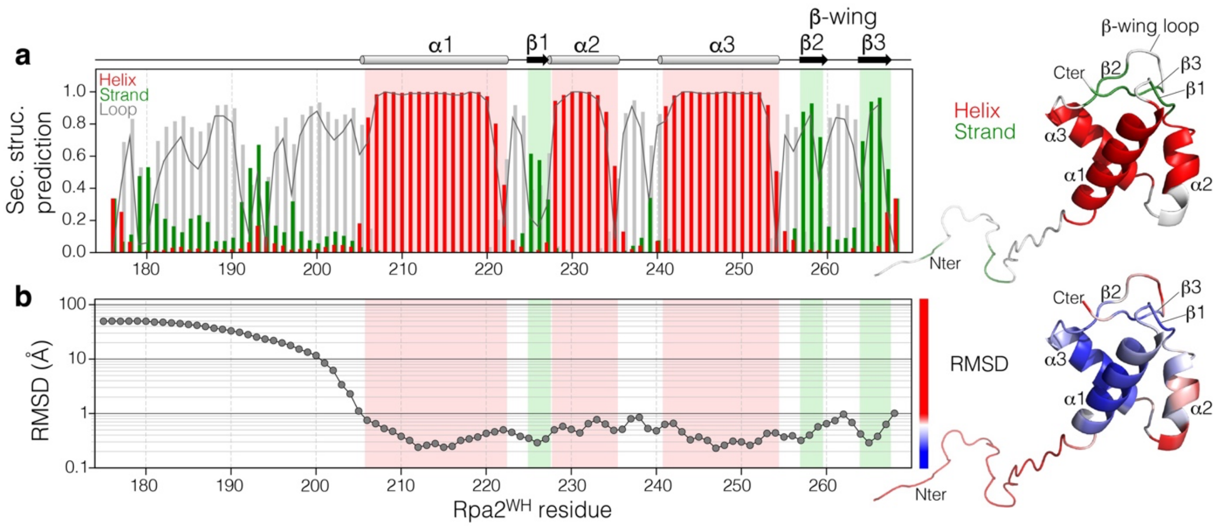

Supplementary Figure 1: **(a)** Secondary structure prediction of Rpa2^{WH} from TALOS-N software based on HN, Hα, Cα, Cβ, CO and N secondary shifts, with predicted helices in red, strands in green and loop/coil regions in grey. The confidence is depicted by the dark grey line. Secondary structure elements inferred from these predictions are indicated at the top. **(b)** Backbone RMSD of the NMR structure ensemble. On the right of each graph, mappings of the secondary structure elements and RMSD are shown on the representative structure of Rpa2^{WH}, with the color coding as indicated. Source data are provided as a Source Data file.

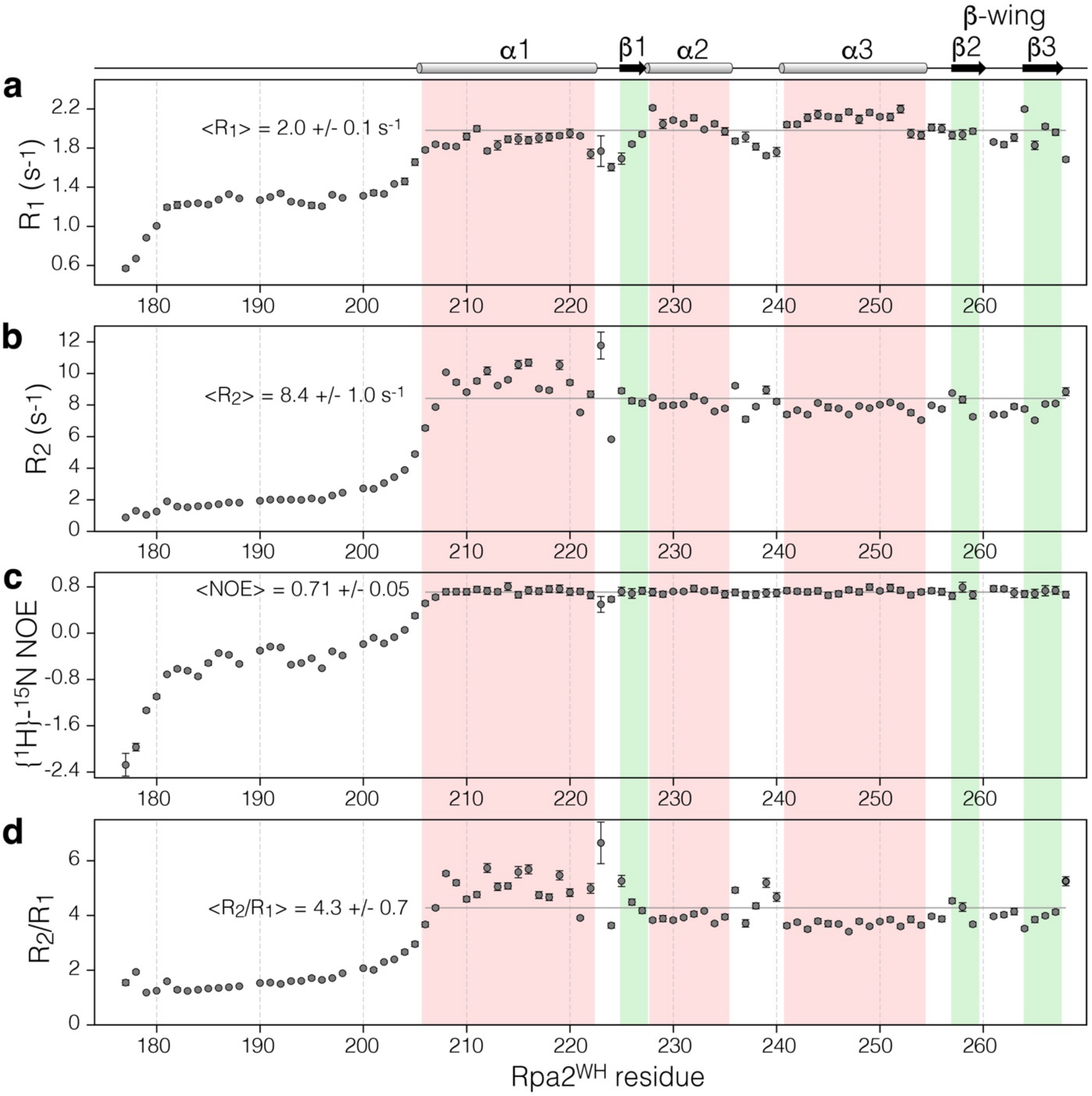

Supplementary Figure 2: **^{15}N relaxation parameters of Rpa2^{WH} at 35°C, 600 MHz**. R_1 **(a)**, R_2 **(b)**, {^{1}H}-^{15}N NOE **(c)** and R_2/R_1 ratio **(d)**. Averages and standard deviations are indicated. Secondary structure elements are displayed at the top; helices and strands are highlighted in red and green, respectively. Source data are provided as a Source Data file.

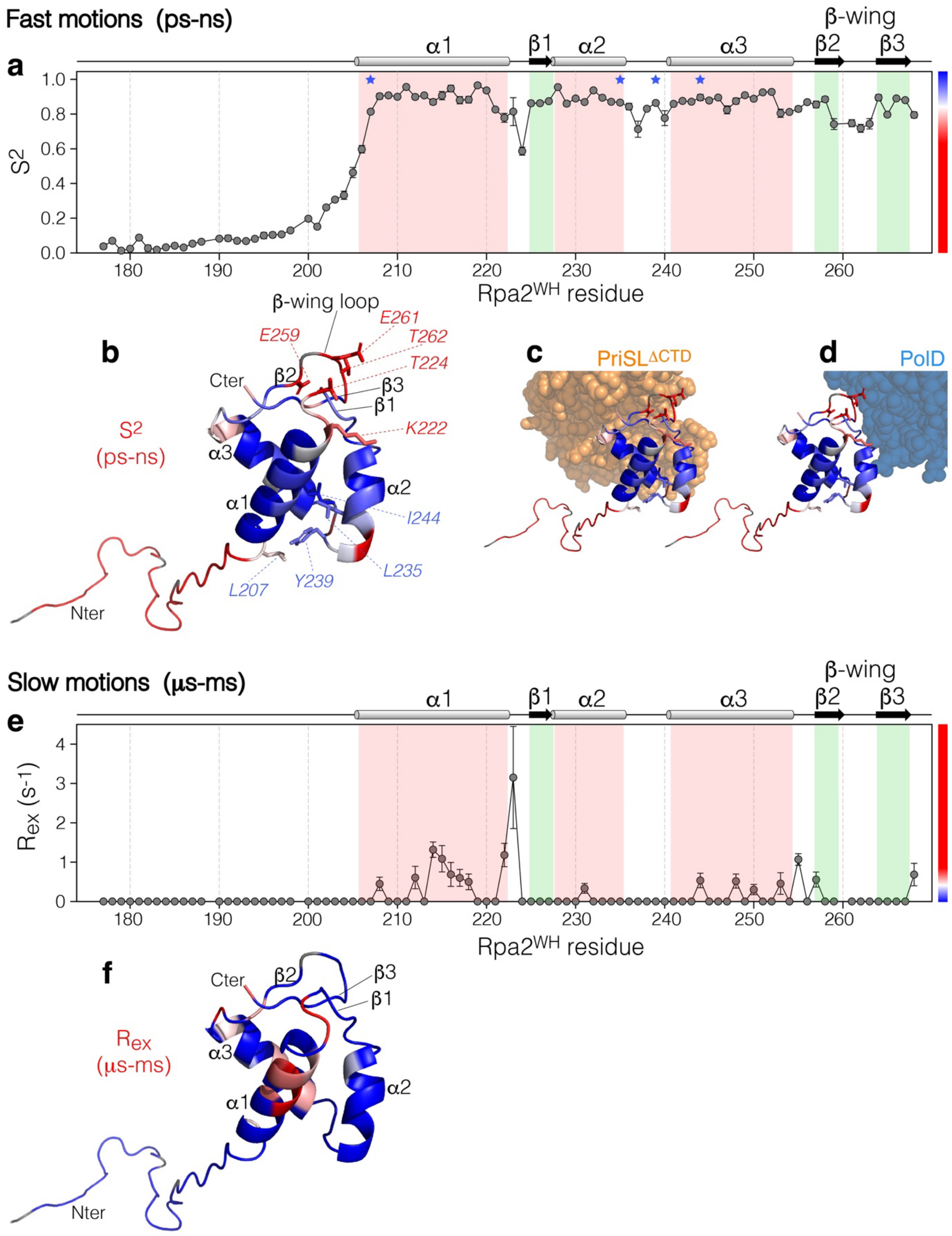

Supplementary Figure 3: Backbone dynamics parameters of Rpa2^{WH}, extracted from ^{15}N relaxation data using the Lipari-Szabo model-free approach with an anisotropic global reorientation model: **(a)** Order parameter S^2 probing motions on the ps-ns timescale. Secondary structure elements are indicated at the top and helices and strands are highlighted in red and green, respectively. **(b)** Fast motions (S^2) mapped on the representative structure of Rpa2^{WH}, with the color coding as indicated on **(a)**, i.e., from blue to red for increasing ps-ns dynamics.

(c, d) Close up view of the binding interface of $Rpa2^{WH}$ with $PriSL^{\Delta CTD}$ (orange spheres) and PolD (blue spheres). **(e)** Exchange contribution R_{ex} probing motions on the µs-ms timescale. **(f)** Slow motions (R_{ex}) mapped on the representative structure of $Rpa2^{WH}$, with the color coding as indicated on **(e)**, i.e., from blue to red for increasing µs-ms dynamics. **Rationalization of the dynamics:** The N-terminal region up to residue 205 is highly flexible. The dynamics of the folded WH domain can be summarized as follows: on the N-terminus side of the domain, the loop between α2/α3 (236-240) has restrained dynamics on the ps-ns timescale due to the stabilization induced by the packing of Y239 into the hydrophobic pocket defined by L207, L235 and I244 (blue asterisks on **(a)** and blue labels on **(b)**), anchoring the N-terminus of α1 to the C-terminus of α2 and to α3 directly after the long flexible N-terminus tail. Opposite to the N-terminus, the region encompassing the loop between α1 and β1 (K222-T224) and the β-wing loop (259-263) define a flexible hotspot (red labels on **(b)**, with $S^2 < 0.75$. Interestingly, this flexible region of $Rpa2^{WH}$ lies against $PriSL^{\Delta CTD}$ **(c)** and PolD **(d)** and is involved in many electrostatic interactions with each partner as observed in the XR and cryo-EM structures of the respective complexes. It is likely that the dynamic nature of this hotspot around the β-wing helps driving fast binding to the cognate polymerases and that this region might be stabilized/rigidified upon complex formation. Conformational exchange in the µs-ms timescale is detected for residues of helices α1 and α3 pointing towards the core of the domain ($R_{ex} \sim 1$ s^{-1}, **(e,f)**). This phenomenon could arise from the propagation of the fast dynamics of the N-terminal tail, slowing down towards the core. Source data are provided as a Source Data file.

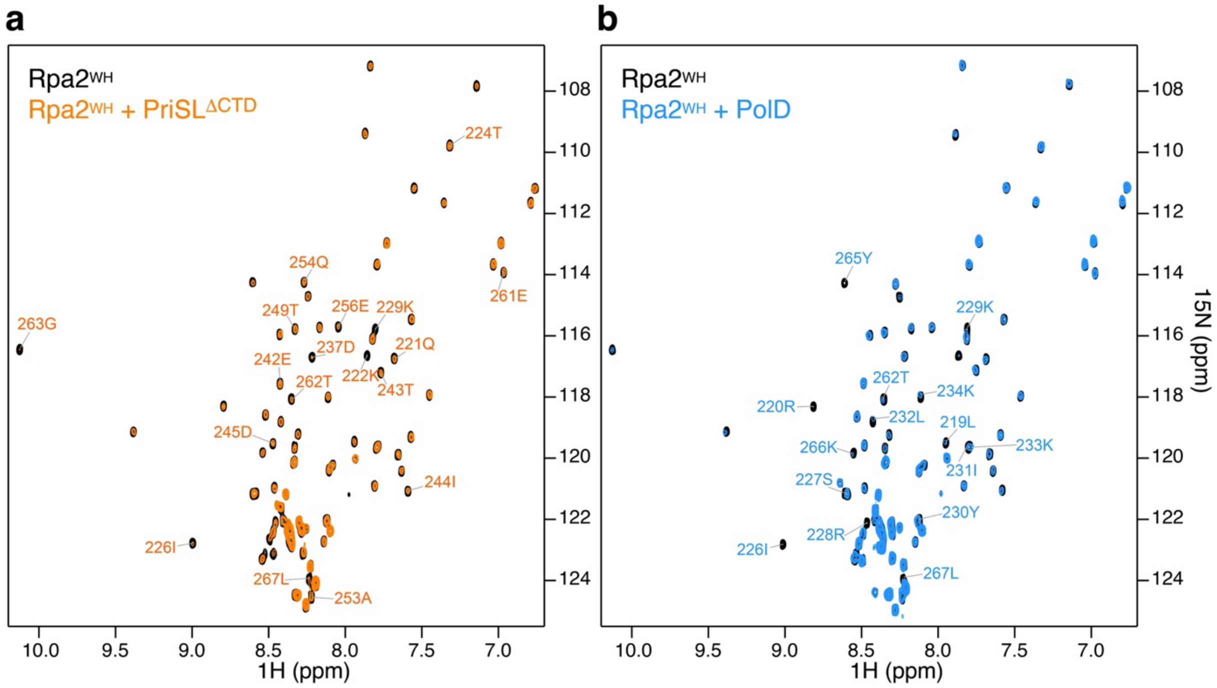

Supplementary Figure 4: Superimposed ^{1}H-^{15}N HSQC spectra recorded on 100 µM ^{15}N-labeled Rpa2^{WH} (**a, b,** black), with 0.5 equivalents of unlabeled PriSL$^{\Delta CTD}$ (**a**, orange) or with 0.1 equivalent of unlabeled PolD (**b**, blue). Assignment is indicated for the most perturbed Rpa2^{WH} signals by PriSL$^{\Delta CTD}$ (I_{cplx}/I_{free} <0.29) or by PolD (I_{cplx}/I_{free} <0.26). These thresholds were used to delineate the respective binding surfaces to the polymerases on **Fig. 3 k, l**.

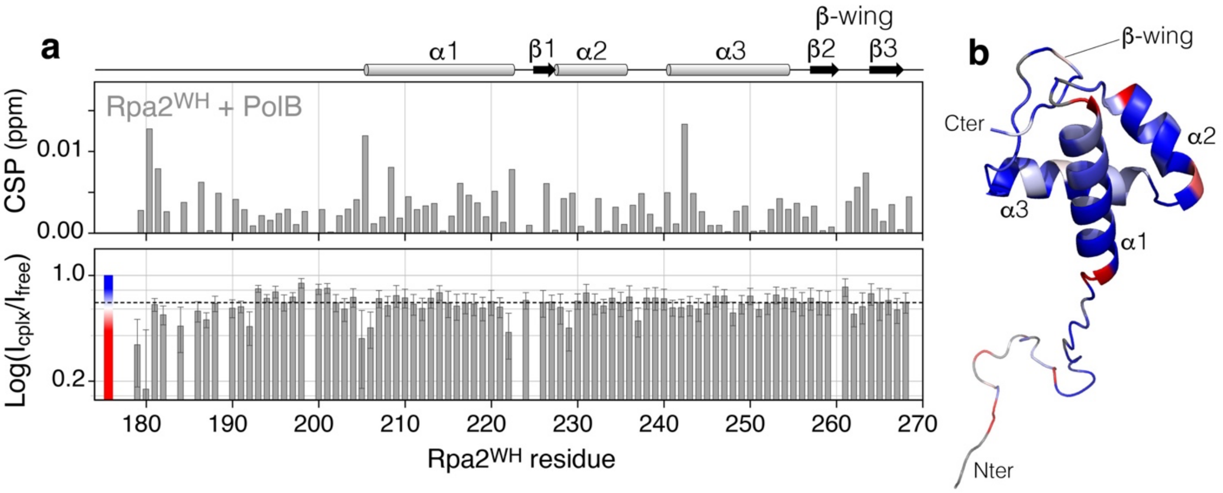

Supplementary Figure 5: **(a)** NMR chemical shift perturbations (CSP) and peak intensity ratios I_{cplx}/I_{free} (log_{10} scale) on $Rpa2^{WH}$ induced by PolB. The average $<I_{cplx}/I_{free}>$ in the folded domain (0.66) is indicated by the dotted line. ^{1}H-^{15}N HSQC spectra were recorded on 50 µM ^{15}N-labeled $Rpa2^{WH}$ with and without an equimolar amount of unlabeled PolB. Secondary structure elements are indicated at the top. **(b)** Mapping of the intensity ratio I_{cplx}/I_{free} on $Rpa2^{WH}$ with the color coding from blue (no attenuation) to red (large attenuation) as indicated. **Rationalization of PolB binding:** Despite an equimolar addition of PolB, the average I_{cplx}/I_{free} ratio (0.66 ± 0.07) in the folded domain is much higher than for the complexes with only 0.5 equivalents of $PriSL^{\Delta CTD}$ (0.32 ± 0.05) or with 0.1 equivalent of PolD (0.31 ± 0.09). In addition, no significant and localized CSP or peak intensity change is detected outside of error bars on $Rpa2^{WH}$ upon addition of PolB. Taken together, these observations indicate that, unlike with the $PriSL^{\Delta CTD}$ and PolD polymerases, no strong and specific binding of the $Rpa2^{WH}$ occurs with PolB. Error bars in the I_{cplx}/I_{free} histograms represent the noise standard deviation in the spectra .Intensity ratios I_{cplx}/I_{free} are calculated from $Rpa2^{WH}$ peak intensities in the complexed (I_{cplx}) and free (I_{free}) forms. Errors on intensity ratios are determined as: $\Delta(I_{cplx}/I_{free}) = I_{cplx}/I_{free} \times ((\Delta I_{cplx}/I_{cplx})^2 + (\Delta I_{ref}/I_{ref})^2)^{1/2}$, where ΔI_{cplx} and ΔI_{free} represent the noise standard deviation in the spectra of complexed and free forms of $Rpa2^{WH}$. Source data are provided as a Source Data File.

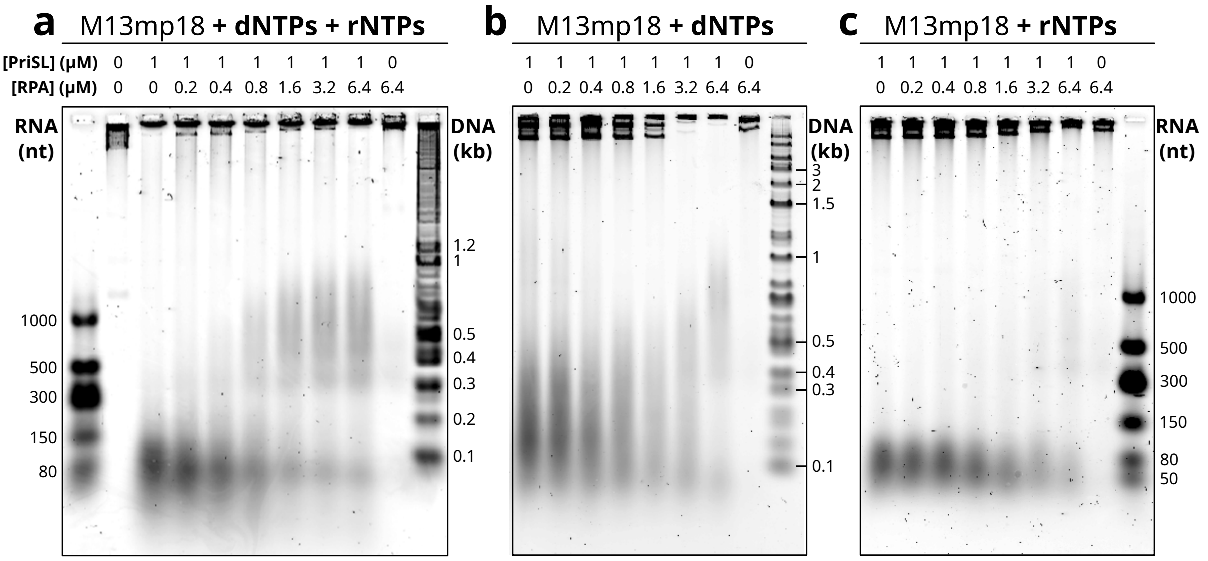

Supplementary Figure 6: **Impact of RPA binding on PriSL priming activity.** M13mp18 circular ssDNA was incubated with PriSL and increasing amounts of RPA (0-6.4 µM) in the presence of dNTPs+rNTPs (**a**), dNTPs only (**b**) or rNTPs only (**c**). Control lanes contain oligonucleotide 1 kb Plus DNA Ladder or Low Range ssRNA Ladder. For details, see the method section. Each experiment was repeated 3 times. Uncropped gels are provided as a Source data File.

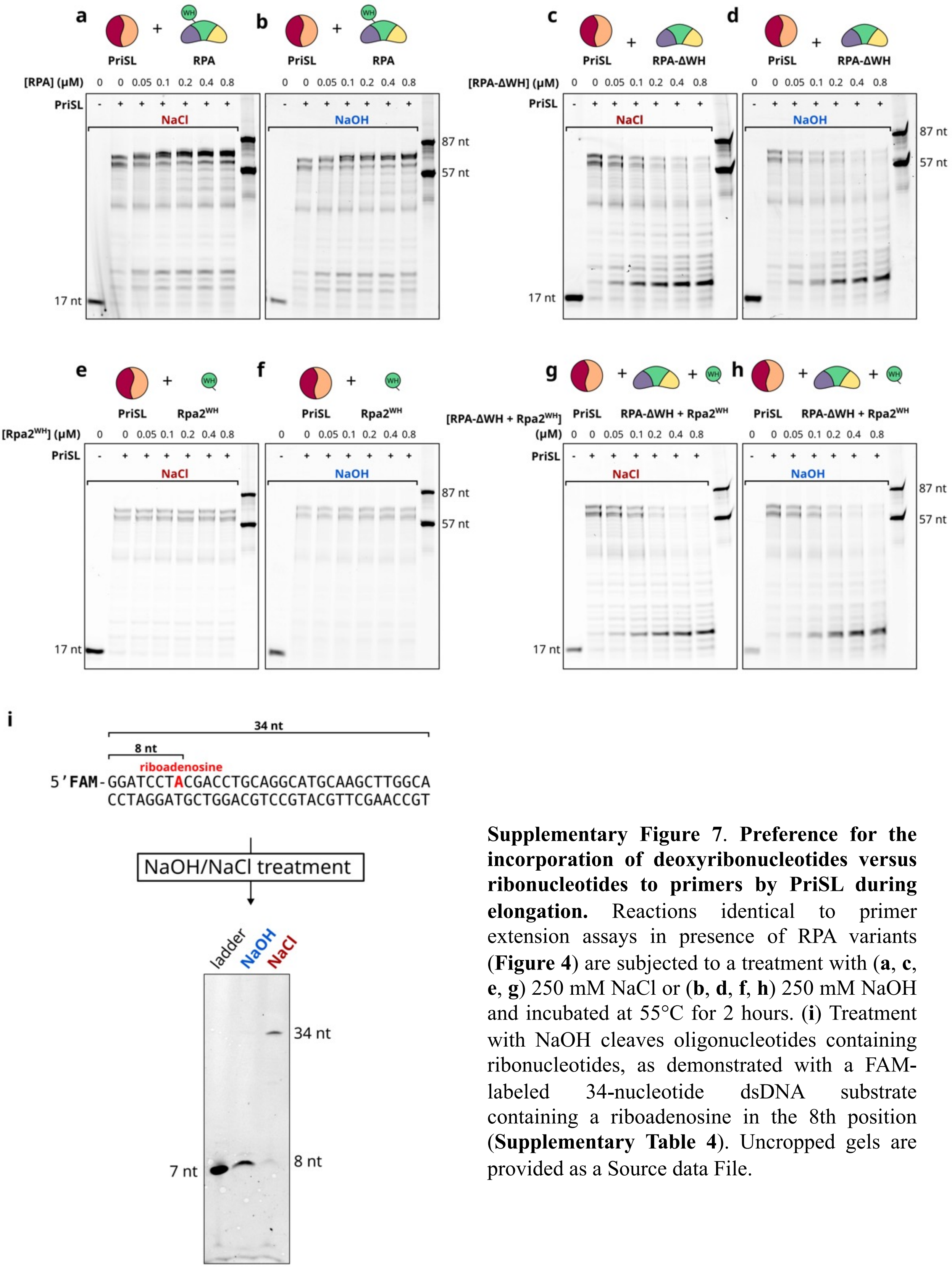

Supplementary Figure 7. Preference for the incorporation of deoxyribonucleotides versus ribonucleotides to primers by PriSL during elongation. Reactions identical to primer extension assays in presence of RPA variants (**Figure 4**) are subjected to a treatment with (**a**, **c**, **e**, **g**) 250 mM NaCl or (**b**, **d**, **f**, **h**) 250 mM NaOH and incubated at 55°C for 2 hours. (**i**) Treatment with NaOH cleaves oligonucleotides containing ribonucleotides, as demonstrated with a FAM-labeled 34-nucleotide dsDNA substrate containing a riboadenosine in the 8th position (**Supplementary Table 4**). Uncropped gels are provided as a Source data File.

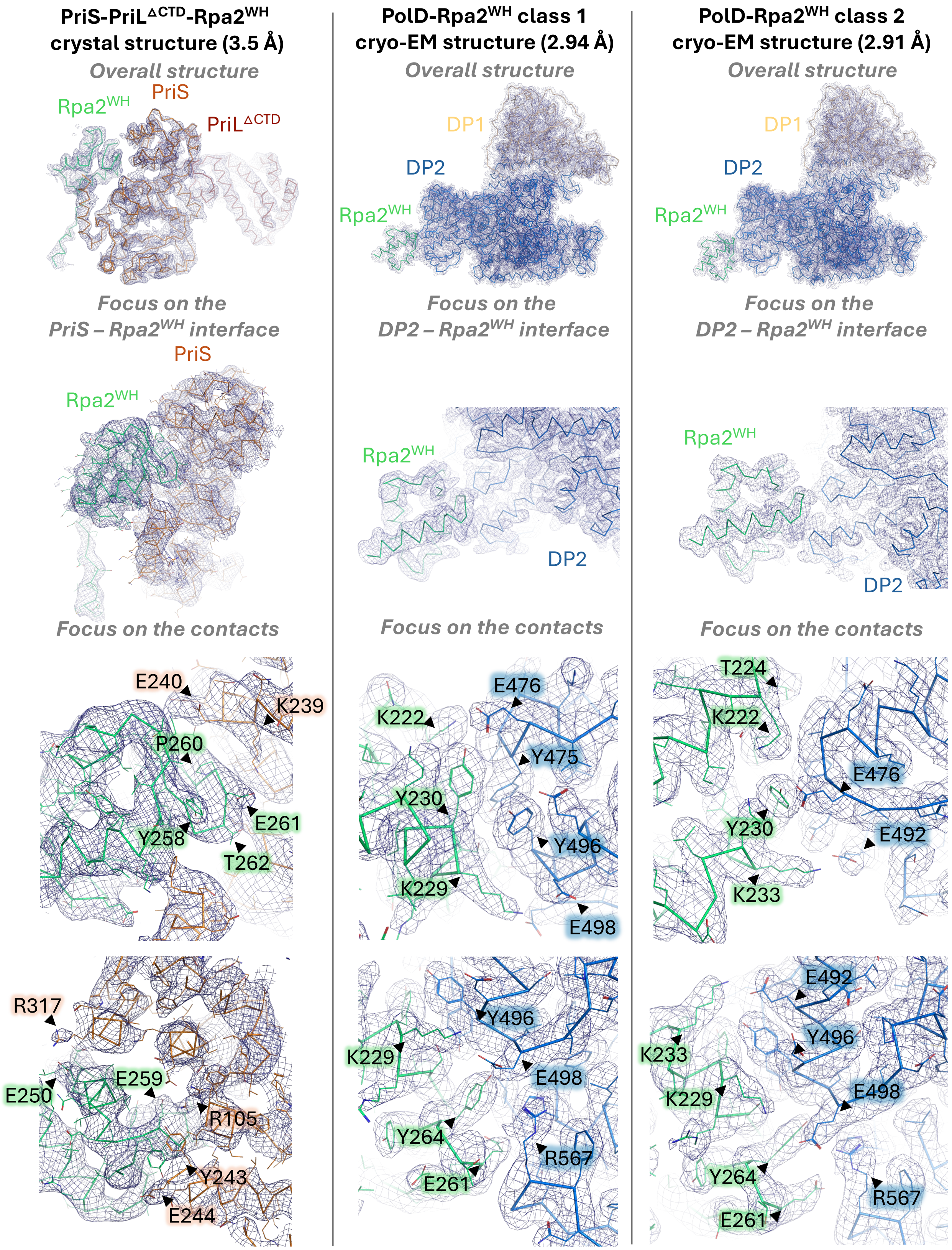

Supplementary Figure 8: Illustration of the quality of the X-ray crystallography electron density map and the cryo-EM maps, in the interfacial region with the Rpa2 winged-helix domain. Left panels show the 2mFo-Fc electron density map contoured at 1.0 σ for the PriS-PriL$^{\Delta CTD}$-Rpa2^{WH} ternary complex. Middle (class1) and right (class2) panels show the cryo-EM maps contoured at a threshold of 0.148 for the DP1-DP2-Rpa2^{WH} ternary complexes.

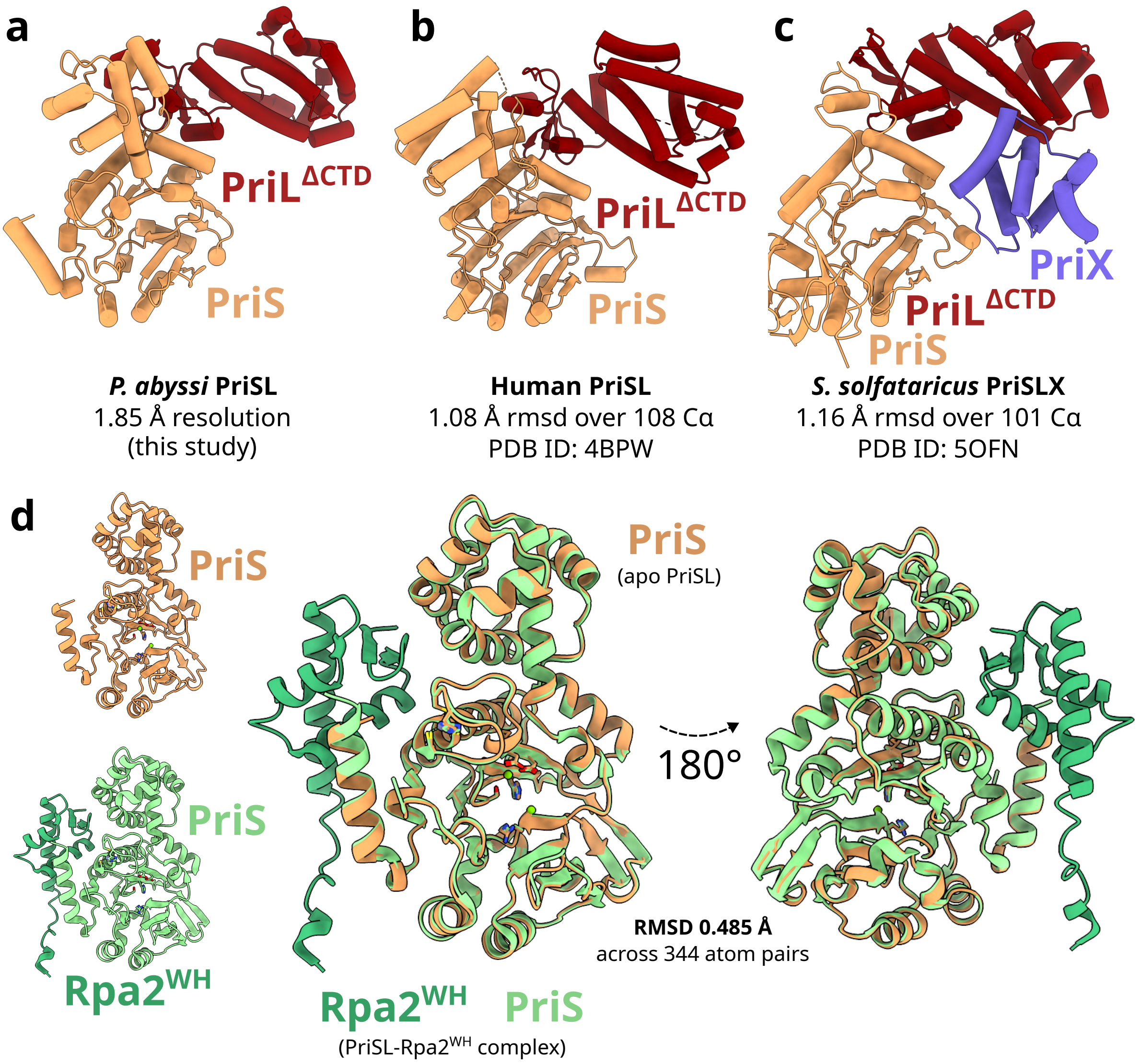

Supplementary Figure 9: **Structural conservation of the heterodimeric euryarchaeal DNA primase.** (**a**) Model of Pyrococcus abyssi PriS-PriL$^{\Delta CTD}$ crystal structure at 1.85 Å resolution (PabPriSL$^{\Delta CTD}$). (**b**) Alignment of human primase (PDB ID 4BPW) to PabPriSL$^{\Delta CTD}$. (**c**) Alignment of Saccharolobus solfataricus PriSLX (PDB ID 5OFN) to PabPriSL$^{\Delta CTD}$. (**d**) Superposition of the crystal structures of PabPriSL$^{\Delta CTD}$ (orange) and the Rpa2WH-PriSL$^{\Delta CTD}$ complex (green), illustrating that PriS does not undergo significant conformational changes upon binding to Rpa2.

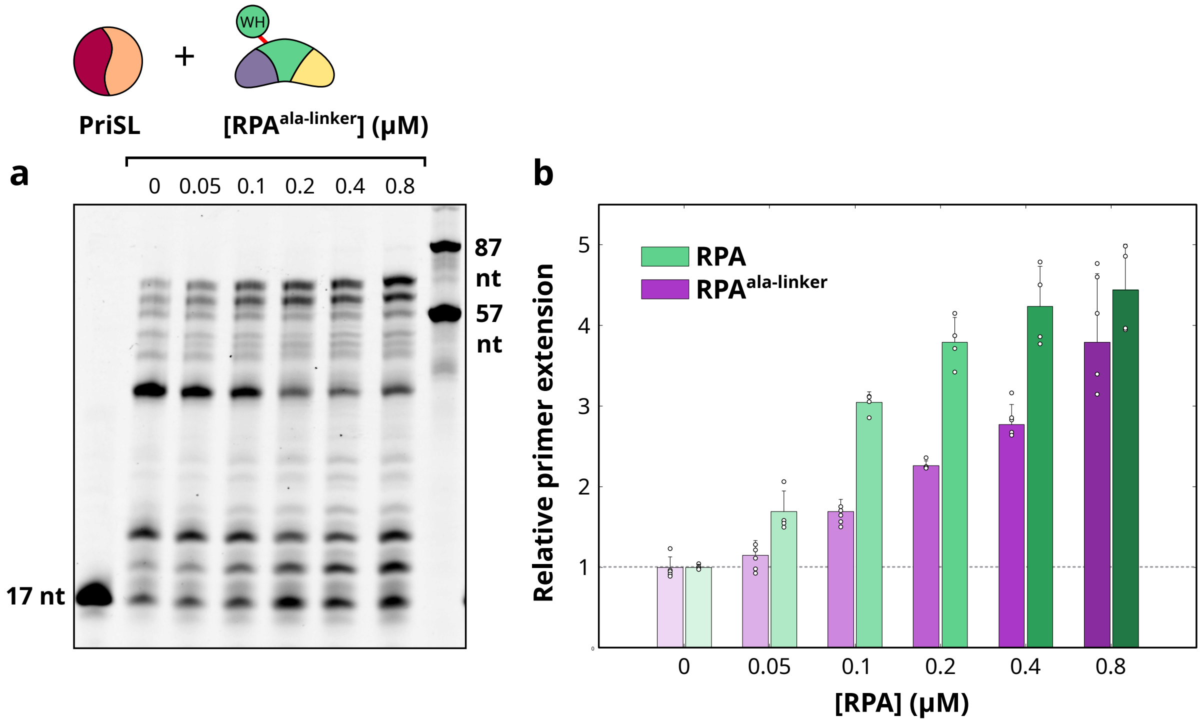

Supplementary Figure 10. Investigating the role of the Rpa2 flexible acidic linker on the ability to stimulate PriSL primer extension activity. (**a**) Primer extension assay by PriSL in the presence of increasing concentrations of $RPA^{ala\text{-}linker}$. (**b**) Comparison of primer extension activity by PriSL in presence of RPA (in green) and $RPA^{ala\text{-}linker}$ (in purple), relative to the activity of PriSL in absence of RPA. Experiments were repeated n=4 times, band integration was performed in all 4 biological replicates (>70 nt bands) to derive standard deviation. Each bar shows the mean value, standard deviation is represented as error bars, and individual measurements are shown as white dots. Uncropped gels are provided as a Source Data file.

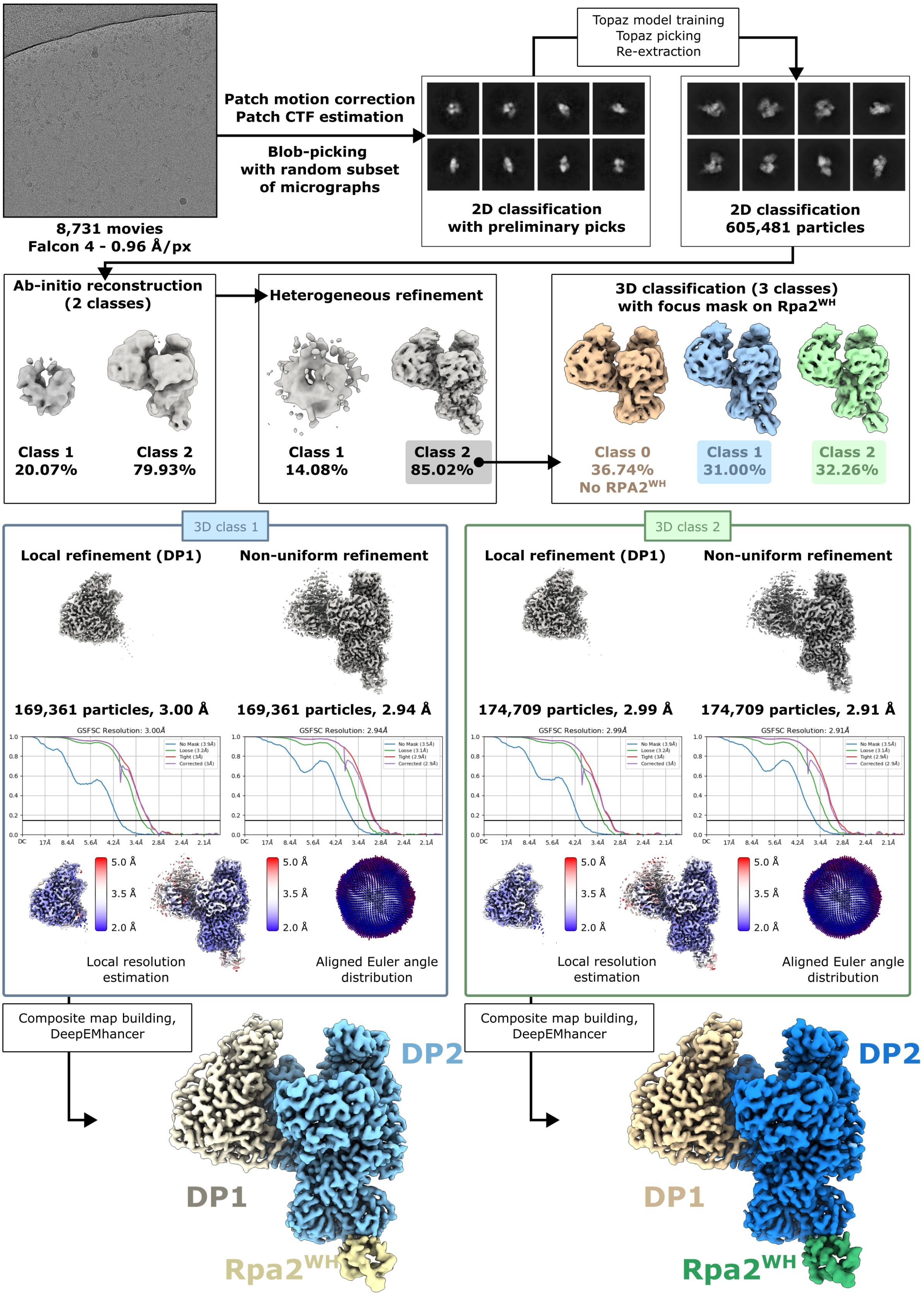

Supplementary Figure 11: Cryo-EM workflow.

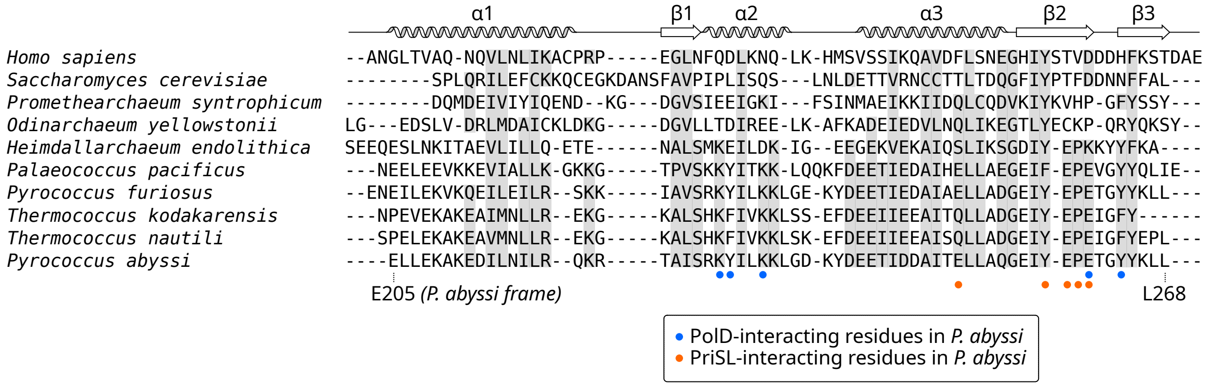

Supplementary Figure 12. Structural alignment of Rpa2 WH domains from archaea and eukaryotic sources. Experimental structures were used when available, and AlphaFold predictions were used for the rest of queries. Sequence conservation is indicated with grey shading. Positions in *P. abyssi* Rpa2 that contact PolD and PriSL are indicated with blue and orange circles respectively.

**Constraints**		**Mean of pairwise RMSD (Å)**	
Dihedral restraints (ϕ)	120	Backbone atoms N, CA, C', O [a]	0.42 ± 0.12
Hydrogen bonds	32	Heavy atoms	1.51 ± 0.14
Distance restraints [a]	954		
**Distance constraints [a,b]**		**Energies (kcal/mol)**	
Unambiguous restraints	685	Total	-3540 ± 120
Ambiguous distance restraints	269	Van der Waals	-342 ± 13.1
Intra-residue | j-i | = 0	494	Electrostatic	-3351 ± 121
Sequential | j-i | = 1	238	**Ensemble Ramachandran plot (% Residues)**	
Medium range 2 ≤ | j-i | ≤ 4	129	Most favored regions	95.9%
Long range | j-i | > 4	93	Additionally allowed	4.1%
**Residual distance constraint violations**			
Number ≥ 0.5 Å	10 ± 1	**Structure Z scores**	
Number ≥ 0.3 Å	12 ± 1	Second-generation packing quality	1.1 ± 0.2
Number ≥ 0.1 Å	18 ± 1	Ramachandran plot appearance	2.2 ± 0.3
**RMS deviation from nOes (Å)**	0.30 ± 0.02	Chi1/Chi2 rotamer normality	1.6 ± 0.8
**Unsatisfied H-bond donors [c]**	4.7	Backbone conformation	-4.7 ± 1.5
**Unsatisfied H-bond acceptors [c]**	0		

Supplementary Table 1. Statistics for the ensemble of 10 structures calculated for full-length Rpa2^{WH}. [a] For well-ordered residues (205-268). [b] For residues 175-204, only 29 intra or sequential distance constraints. [c] Per molecule. PDB ID: 9F27; BMRB ID: 34913.

	$PriSL^{\Delta CTD}$	$PriSL^{\Delta CTD}$+$Rpa2^{WH}$
**Data collection**		
Space group	*P* $4_3 2_1 2$	*P* $2_1 2_1 2_1$
Cell dimensions		
a, *b*, *c* (Å)	116.6, 116.6, 121.1	68.8, 101.5, 177.8
α, β, γ (°)	90, 90, 90	90, 90, 90
Wavelength (Å)	0.98011	0.97857
Resolution (Å)	39.5 - 1.85 (1.89 - 1.85)	48.8 - 3.50 (3.59 - 3.50)
Estimated resolution limit (Å)*	1.94, 1.94, 1.84	3.15, 3.59, 5.93
R_{pim}	0.023 (0.609)	0.036 (4.24)
R_{merge}	0.114 (4.06)	0.126 (15.0)
$I / \sigma I$	12.6 (0.8)	9.5 (0.3)
$CC_{1/2}$	0.999 (0.592)	1.0 (0.20)
Completeness (%)	100 (100)	99.9 (99.5)
Redundancy	26.1 (24.8)	13.2 (13.4)
R_{pim}*	0.025 (0.427)	0.022 (0.506)
R_{merge}*	0.125 (2.14)	0.074 (1.774)
$I / \sigma I$*	14.0 (1.5)	16.1 (1.8)
$CC_{1/2}$*	0.999 (0.745)	1.000 (0.593)
Completeness (%)*	96.4 (61.5)	58.1 (16.0)
**Refinement**		
Resolution (Å)	39.51 - 1.85	48.8 - 3.50 (3.67-3.50)
No. reflections	67117 (1284)	8875 (386)
R_{work} / R_{free} (%)	19.87/22.37	23.45/25.55
No. atoms		
Protein	4606	5289
Ligand/ion	9	1
Water	611	-
B-factors		
Protein	43.1	184.1
Ligand/ion	54.3	44.13
Water	57.0	-
R.m.s. deviations		
Bond lengths (Å)	0.009	0.0085
Bond angles (°)	0.89	1.32
PDBID	9F28	9F26

Supplementary Table 2. Crystallographic and refinement statistics. Values in parentheses are for highest-resolution shell. Dataset from single crystal used per structure. Values calculated after truncation by STARANISO (*). Estimated resolution limits along the three crystallographic directions a*, b*, c*.

Data collection and processing	**PolD-Rpa^{WH} class 1** EMDB-50140 PDB 9F29	**PolD-Rpa^{WH} class 2** EMDB-50143 PDB 9F2A
Magnification	130,000X	130,000X
Voltage (kV)	300	300
Electron exposure ($e^-/Å^2$)	40	40
Pixel size (Å)	0.96	0.96
Symmetry imposed	-	-
Initial particle images (no.)	605,481	605,481
Final particle images (no.)	169,361	174,709
Map resolution (Å) (FSC threshold)	2.94 (0.143)	2.91(0.143)
Map resolution range (Å)	2-5	2-5
**Refinement**		
Initial model used (PDB code)	6T8H	6T8H
Model resolution (Å) (FSC threshold)	2.8 (0.5)	2.9 (0.5)
Model resolution range (Å)	2-5	2-5
Map sharpening *B* factor ($Å^2$)	97.2	96.7
Model composition		
Non-hydrogen atoms	13520	13557
Protein residues	1688	1692
Ligands	4	4
B factors ($Å^2$)		
Protein	58.57	58.79
Ligand	101.13	116.38
RMS deviations		
Bond lengths (Å)	0.003	0.004
Bond angles (°)	0.488	0.549
Validation		
Molprobity score	1.38	1.24
Clashscore	3.38	3.12
Poor rotamers (%)	0	0
Ramachandran plot		
Favored (%)	96.3	97.26
Allowed (%)	3.7	2.74
Disallowed (%)	0	0

Supplementary Table 3. Cryo-EM data collection, refinement and validation statistics.

Oligonucleotides	Length (nt)	Séquences (5' to 3')	Label
**Primer**	17	TGCCAAGCTTGCATGCC	5'Cy5
**Template**	87	CAGGAAACAGCTATGACCATGATTACGAATTCGAGCTCGGTACCCGGGGATCCTCTAGAGTCGACCTGCAGGCATGCAAGCTTGGCA	
**Ladders**	87	TGCCAAGCTTGCATGCCTGCAGGTCGACTCTAGAGGATCCCCGGGTACCGAGCTCGAATTCGTAATCATGGTCATAGCTGTTTCCTG	5'Cy5
	57	TGCCAAGCTTGCATGCCTGCAGGTCGACTCTAGAGGATCCCCGGGTACCGAGCTCGA	5'Cy5
**Oligonucleotide competitor**	87	TGCCAAGCTTGCATGCCTGCAGGTCGACTCTAGAGGATCCCCGGGTACCGAGCTCGAATTCGTAATCATGGTCATAGCTGTTTCCTG	
**Forward**	34	GGATCCTaCGACCTGCAGGCATGCAAGCTTGGCA	5'FAM
**reverse**	34	TGCCAAGCTTGCATGCCTGCAGGTCGTAGGATCC	

Supplementary Table 4. Oligonucleotides used as ladders and primer-templates in the activity assay experiments. Deoxyribonucleotides are in capital letters and ribonucleotide is in lower case letter.

Strains	Genotype or other relevant characteristics	Source or reference
E. coli		
DH5α	*Φ80dlacZΔm15, recA1, endA1, gyrA96, thi-1, hsdR17 (r_k−, m_k+), supE44, relA1, deoR, Δ(lacZYA-argF)U169*	Thermo Fisher Scientific
T. barophilus		
UBOCC-M-3300	Δ*TERMP_00517*	(Birien et al., 2018)
**Plasmids**		
pUPH	Pop-in Pop-out vector	(Birien et al., 2018)
pRD603	pUPH-RPAΔ70AA Cter	This study
pRD605	pUPH-RPAΔ3SU	This study

Supplementary Table 5: Strains and plasmids used in this study.

**761-RPA32ΔWHCterKpnI**	ATCG**GGTACC**AGATCAGGCGTAGAAAGCCAGG	To construct RPAΔ^{WH}
**762-RPA32ΔWHCterFusRv**	CCAATTTCTTTTTTACAAAAAGAGGAATAAGAAGTCATTCTTCCTCAAATATTTCCTCCTCTAAGGC	
**763-RPA32ΔWHCterFusFw**	GCCTTAGAGGAGGAAATATTTGAGGAAGAATGACTTCTTATTCCTCTTTTTGTAAAAAAGAAATTGG	
**764-RPA32ΔWHCterBamHI**	GCTA**GGATCC**GGCTCAACATATATGCGTTTATTCTGGC	
**766-RPA32ΔWHVerifRv**	GGTATGCGATGCTCTTATTTGTTGTTGG	Used with 776. To verify mutant of RPA
**771-RPA-SupBamHI-Fw**	CCCTGCACTTATCCCCGAGAATCCATTTTCCAAGGATTATCTTCTCC	To suppress *Bam*HI site in the sequence encoding RPA
**772-RPA-SupBamHI-Rv**	GGAGAAGATAATCCTTGGAAAATGGATTCTCGGGGATAAGTGCAGGG	
**773-RPAΔ3SU-KpnI**	ATGC**GGTACC**AGTGACAGTCCCGCATGATAGG	To delete the three RPA genes
**774-RPAΔ3SU-FusRv**	GGTCATTTTGCAAAATCTGGAGCCTTCTTATTCCTCTTTTTGTAAAAAAGAAATTGG	
**775-RPAΔ3SU-FusFw**	CCAATTTCTTTTTTACAAAAAGAGGAATAAGAAGGCTCCAGATTTTGCAAAATGACC	
**776-RPAΔ3SUVerifFw**	GGGTTAGTATTCTAATTTTACCTCTCTTCAAAGCG	Used with 766. To verify mutant of RPA

Supplementary Table 6: List of primers used in this study. Restriction sites are shown in bold.
