# Supplementary material for: Communication between DNA polymerases and Replication Protein A within the archaeal replisome
Source: Nat Commun. 2024 Dec 30;15:10926. doi: 10.1038/s41467-024-55365-w (PMC11686378; doi:10.1038/s41467-024-55365-w)
Supplement: Supplementary file 2 — Reporting Summary [file 41467_2024_55365_MOESM2_ESM.pdf]

nature portfolio

Corresponding author(s): Ludovic Sauguet, Ghislaine Henneke

Last updated by author(s): Nov 28, 2024

Reporting Summary

Nature Portfolio wishes to improve the reproducibility of the work that we publish. This form provides structure for consistency and transparency in reporting. For further information on Nature Portfolio policies, see our Editorial Policies and the Editorial Policy Checklist.

Statistics

For all statistical analyses, confirm that the following items are present in the figure legend, table legend, main text, or Methods section.

n/a	Confirmed	
☐	☒	The exact sample size (*n*) for each experimental group/condition, given as a discrete number and unit of measurement
☐	☒	A statement on whether measurements were taken from distinct samples or whether the same sample was measured repeatedly
☒	☐	The statistical test(s) used AND whether they are one- or two-sided<br>*Only common tests should be described solely by name; describe more complex techniques in the Methods section.*
☒	☐	A description of all covariates tested
☒	☐	A description of any assumptions or corrections, such as tests of normality and adjustment for multiple comparisons
☐	☒	A full description of the statistical parameters including central tendency (e.g. means) or other basic estimates (e.g. regression coefficient) AND variation (e.g. standard deviation) or associated estimates of uncertainty (e.g. confidence intervals)
☒	☐	For null hypothesis testing, the test statistic (e.g. *F*, *t*, *r*) with confidence intervals, effect sizes, degrees of freedom and *P* value noted<br>*Give P values as exact values whenever suitable.*
☒	☐	For Bayesian analysis, information on the choice of priors and Markov chain Monte Carlo settings
☒	☐	For hierarchical and complex designs, identification of the appropriate level for tests and full reporting of outcomes
☒	☐	Estimates of effect sizes (e.g. Cohen's *d*, Pearson's *r*), indicating how they were calculated

Our web collection on statistics for biologists contains articles on many of the points above.

Software and code

Policy information about availability of computer code

Data collection	EPU (thermofischer) was used for Cryo-EM data collection
Data analysis	For all structural biology data analysed in the context of this work, the following software were used: COOT v0.9.6, AlphaFold2 v2.3, Cryosparc v.4.2, Phenix v1.19-4092, APBS v1.4, DALI v5, UCSF Chimera X v1.0. All these tools are described in the literature and publicly available.

For manuscripts utilizing custom algorithms or software that are central to the research but not yet described in published literature, software must be made available to editors and reviewers. We strongly encourage code deposition in a community repository (e.g. GitHub). See the Nature Portfolio guidelines for submitting code & software for further information.

Data

Policy information about availability of data

All manuscripts must include a data availability statement. This statement should provide the following information, where applicable:

- Accession codes, unique identifiers, or web links for publicly available datasets
- A description of any restrictions on data availability
- For clinical datasets or third party data, please ensure that the statement adheres to our policy

Cryo-EM: Individual component maps of the DP1 domain and DP2-Rpa2WH from 3D class 1 are available on the EMDB as entries 50141 and 50142 respectively. Individual component maps of the DP1 domain and DP2-Rpa2WH from 3D class 2 are available on the EMDB as entries 50144 and 50145 respectively. Coordinates and composite maps of 3D classes 1 and 2 are available as PDB entries 9F29 and 9F2A and EMDB entries 50140 and 50143 respectively. NMR: Coordinates and chemical shifts of Rpa2WH are deposited on the PDB (9F27) and BMRB (34913) repositories, respectively. X-ray crystallography: Coordinates and structure factors of

the PriS-PriL(1–210) and PriS-PriL(1–210)-RPA2(190-268) crystal structures from P. abyssi were deposited in the Protein Data Bank under accession codes 9F28 and 9F26, respectively.

Research involving human participants, their data, or biological material

Policy information about studies with human participants or human data. See also policy information about sex, gender (identity/presentation), and sexual orientation and race, ethnicity and racism.

Reporting on sex and gender	N/A
Reporting on race, ethnicity, or other socially relevant groupings	N/A
Population characteristics	N/A
Recruitment	N/A
Ethics oversight	N/A

Note that full information on the approval of the study protocol must also be provided in the manuscript.

Field-specific reporting

Please select the one below that is the best fit for your research. If you are not sure, read the appropriate sections before making your selection.

[x] Life sciences   [ ] Behavioural & social sciences   [ ] Ecological, evolutionary & environmental sciences

For a reference copy of the document with all sections, see nature.com/documents/nr-reporting-summary-flat.pdf

Life sciences study design

All studies must disclose on these points even when the disclosure is negative.

Sample size	Binding experiments that were performed in this work usually involve 4000 points. To determine the binding constants, binding experiments were performed at 7 different concentrations, and replicated as detailed in the figure legends (>=4). All replicates are biological replicates not technical replicates.
Data exclusions	No data were excluded.
Replication	All attempts at replication were succesfull.
Randomization	For binding studies, randomization of the data is not easily applicable. For an optimal binding experiments, the concentrations of the ligands must be selected in a concentration range that covers the dissociation constant.
Blinding	Blinding, which refers to the concealment of group allocation from one or more individuals involved in a clinical research study is not easily applicable to our biophysical experiments. Indeed, identifying the target protein and the control is important for the technical set-up of these experiments. For example, this enables us to perform the binding of the target protein at multiple concentrations simultaneously.

Reporting for specific materials, systems and methods

We require information from authors about some types of materials, experimental systems and methods used in many studies. Here, indicate whether each material, system or method listed is relevant to your study. If you are not sure if a list item applies to your research, read the appropriate section before selecting a response.

Materials & experimental systems

n/a	Involved in the study	
[x]	[ ]	Antibodies
[x]	[ ]	Eukaryotic cell lines
[x]	[ ]	Palaeontology and archaeology
[x]	[ ]	Animals and other organisms
[x]	[ ]	Clinical data
[x]	[ ]	Dual use research of concern
[x]	[ ]	Plants

Methods

n/a	Involved in the study	
[x]	[ ]	ChIP-seq
[x]	[ ]	Flow cytometry
[x]	[ ]	MRI-based neuroimaging
